# Development of dried tube specimens for Xpert MTB/RIF proficiency testing

**DOI:** 10.4102/ajlm.v9i1.1166

**Published:** 2020-09-29

**Authors:** Kyle DeGruy, Katherine Klein, Zilma Rey, Patricia Hall, Andrea Kim, Heather Alexander

**Affiliations:** 1 Centers for Disease Control and Prevention, Division of Global HIV and TB, International Laboratory Branch, Atlanta, Georgia, United States; 2 Centers for Disease Control and Prevention, Division of TB Elimination, Laboratory Branch, Atlanta, Georgia, United States

**Keywords:** external quality assessment, EQA, Xpert MTB/RIF, proficiency testing, dried tube specimen, DTS

## Abstract

**Background:**

Proficiency testing (PT) is part of a comprehensive quality assurance programme, which is critical to ensuring patients receive accurate and reliable diagnostic testing. Implementation of the Cepheid Xpert® MTB/RIF assay to aid in the diagnosis of tuberculosis has expanded rapidly in recent years; however, PT material for Xpert MTB/RIF is not readily available in many resource-limited settings.

**Objective:**

To develop an accurate and precise PT material based on the dried tube specimen (DTS) method, using supplies and reagents available in most tuberculosis culture laboratories.

**Methods:**

Dried tube specimens were produced at the United States Centers for Disease Control and Prevention from 2013 to 2015 by inactivating liquid cultures of well-characterised mycobacterial strains. Ten percent of DTS produced were tested with Xpert MTB/RIF and evaluated for accuracy and precision.

**Results:**

Validation testing across eight rounds of PT demonstrated that DTS are highly accurate, achieving an average of 96.8% concordance with the Xpert MTB/RIF results from the original mycobacterial strains. Dried tube specimen testing was also precise, with cycle threshold standard deviations below two cycles when inherent test cartridge variability was low.

**Conclusion:**

Dried tube specimens can be produced using equipment already present in tuberculosis culture laboratories, making Xpert MTB/RIF PT scale-up more feasible in resource-limited settings. Use of DTS may fill the gap in tuberculosis laboratory access to external quality assessment, which is an essential component of a comprehensive continuous quality improvement programme.

## Introduction

The World Health Organization (WHO) estimated that 10.0 million people developed tuberculosis and 1.5 million died from tuberculosis disease worldwide in 2018.^1^ Human immunodeficiency virus contributes to this epidemic, with 860 000 (8.6%) of the 10.0 million tuberculosis patients also being co-infected with HIV. Approximately 251 000 people living with human immunodeficiency virus died from tuberculosis in 2018.^1^ Rapid tuberculosis diagnosis and treatment initiation are therefore critical to reducing tuberculosis-related deaths and ongoing transmission in communities.

Access to rapid, quality diagnostic testing is a significant barrier to global tuberculosis elimination.^2^ The WHO estimated that approximately 3 million cases of tuberculosis disease in 2018 went undiagnosed or unreported, and only 186 772 of the estimated 500 000 people who developed either multidrug-resistant or rifampicin-resistant tuberculosis were diagnosed.^1^ The implementation of liquid culture and molecular methods have shortened the time-to-detection of *Mycobacterium tuberculosis* and time-to-determination of the *M. tuberculosis* drug susceptibility profile.^3,4^ The Cepheid Xpert® MTB/RIF assay (Cepheid, Sunnyvale, California, United States) is a cartridge-based, hemi-nested, real-time polymerase chain reaction assay detecting *M. tuberculosis* and genetic mutations associated with resistance to rifampicin.^5^ This assay provides test results within two hours of beginning the test when compared to conventional culture and phenotypic drug susceptibility testing, which may take eight weeks or longer.^5,6^ The WHO recommends Xpert MTB/RIF as one of the initial diagnostic tests for all individuals evaluated for tuberculosis.^7^

Quality assurance (QA) is vital in the clinical laboratory for accuracy and reliability of diagnostic testing. Clinicians depend on accurate and rapid results for appropriate patient diagnosis and treatment.^8^ Rapid implementation of the Xpert MTB/RIF assay occurred in many resource-limited settings to quickly increase capacity for tuberculosis diagnosis and treatment initiation; unfortunately, QA activities have not kept pace with the expansion of Xpert MTB/RIF testing. When we initiated development of the dried tube specimen (DTS) for the Xpert MTB/RIF external quality assessment (EQA) procedure in 2012, only 386 of 907 (43%) global Xpert MTB/RIF testing sites participated in EQA, and only 77 of 318 (24%) African testing sites, participated in EQA.^9^ Quality assessment programmes for Xpert MTB/RIF are needed to ensure patients receive accurate tuberculosis diagnostics, rifampicin resistance testing, and follow-up test results. A comprehensive QA plan for Xpert MTB/RIF includes documented training and competency assessment for all operators, verification of the GeneXpert instrument or modules, new lot testing, temperature monitoring for testing site and kit storage, routine monitoring of quality indicators, and EQA, including onsite supervisory visits and participation in proficiency testing (PT).^10,11^ Proficiency testing provides opportunities for the laboratory to identify errors in the testing process and implement systems to detect and prevent those errors.^12^

Although a limited number of Xpert MTB/RIF PT panels are currently available to testing sites in resource-limited settings, many must be procured from commercial international providers.^13^ This may be cost prohibitive, and transport of infectious material can be complicated.^14^ Because of these constraints, as well as the rapidly expanding number of testing sites, there is a critical need for countries to implement a simple and sustainable national PT programme in order to ensure the accuracy of Xpert MTB/RIF results. We modified the DTS method originally developed for HIV rapid test PT to produce PT panels for the Xpert MTB/RIF assay.^15^ The novel DTS-based Xpert MTB/RIF PT panel methodology described here was developed to address the need for countries to sustainably produce their own PT panels for the Xpert MTB/RIF assay.

## Methods

### Ethical considerations

No patient specimen collection was required, and no patient identifiers were recorded on quality assurance tools. Participation in the Xpert MTB/RIF quality assurance and PT programme was voluntary and free of charge. No incentives were provided. This study was approved by the CDC Center for Global Health, Division of Human Research Protection (CGH HSR tracking # 2014-082).

### Dried tube specimen panel preparation

Dried tube specimens for this study were prepared at the United States Centers for Disease Control and Prevention (CDC; Atlanta, Georgia, United States), International Laboratory Branch between 2013 and 2015. The Xpert MTB/RIF cartridge captures and lyses intact *M. tuberculosis* bacilli.^5^ Dried tube specimens were developed using whole cell inactivation in order to preserve the cell structure of the selected mycobacterial strains. This more closely simulates DNA extraction from a patient specimen. All DTS preparation steps were performed in a Biosafety Level 3 containment laboratory with personal protective equipment, including respiratory protection. Dried tube specimens were prepared using well-characterised rifampicin-resistant and rifampicin-susceptible *M. tuberculosis* strains and non-tuberculous mycobacteria strains obtained from the American Type Culture Collection, WHO proficiency testing challenges, and the CDC’s collection of laboratory-derived *M. tuberculosis* strains. *M. tuberculosis* strains were characterised using phenotypic and genotypic methods, including the Middlebrook 7H10 method of proportion drug susceptibility testing and Sanger sequencing of the *rpoB* gene.^6,16^ Strains were inoculated into PANTA-supplemented BACTEC® MGIT (Mycobacteria Growth Indicator Tube) 7 mL tubes (Modified Middlebrook 7H9 broth base) (Becton, Dickinson and Company; Sparks, Maryland, United States) and incubated in the BACTEC® MGIT 960® instrument (Becton, Dickinson and Company; Sparks, Maryland, United States) until flagged positive for culture growth. Cultures were then incubated an additional 4–6 days at 37 °C in an auxiliary incubator and inoculated onto Middlebrook 7H10 agar and blood agar (Remel, Lenexa, Kansas, United States) for enumeration of mycobacteria and contamination checks.

**FIGURE 1:**
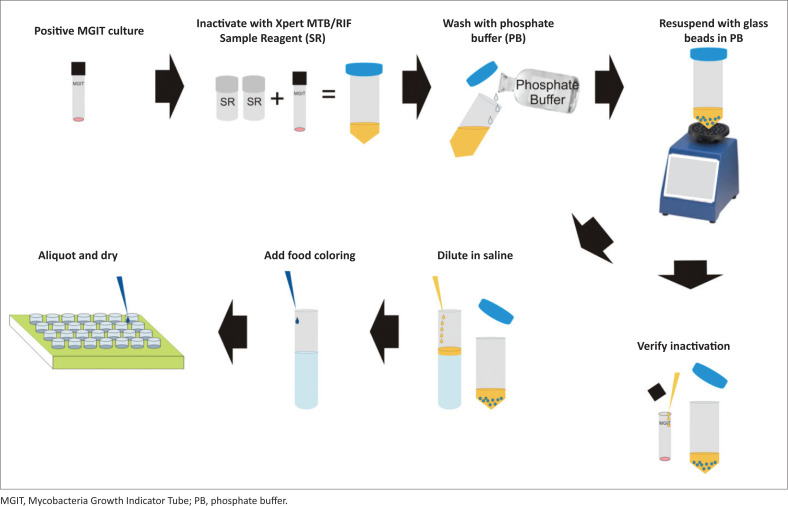
Overview of dried tube specimens for Xpert MTB/RIF preparation procedure, 2013–2015, United States Centers for Disease Control and Prevention (Atlanta, Georgia, United States).

Mycobacteria were inactivated using a 2:1 ratio of Xpert MTB/RIF Sample Reagent (SR) (Cepheid; Sunnyvale, California, United States) to MGIT culture in sterile 50 mL conical tubes (Figure 1). The suspensions were incubated at 15 °C – 30 °C, and vortexed for 30 seconds every 15 minutes for a total of two hours. MGIT culture/SR suspensions were neutralised with 20 mL – 25 mL of in-house prepared, sterile phosphate buffer pH 6.8 and centrifuged at 3000 × g for 15 min. The pellets were washed with an additional 45 mL phosphate buffer, centrifuged, and the supernatants were discarded. Pellets were then resuspended in 5 mL of phosphate buffer, transferred to sterile glass tubes with 5–10 3 mm glass beads (Thermo Fisher Scientific, Waltham, Massachusetts, United States), vortexed for 5 min, and allowed to settle for 15 min. Supernatants above the beads were transferred to new sterile glass tubes, labelled as stock suspensions, and stored at 2 °C – 8 °C. Inactivation verification was performed by inoculating 0.5 mL of stock suspension into MGIT tubes supplemented with PANTA and incubating for two cycles for a total of 84 days. Only stocks testing negative for growth were considered for panel preparation and distribution.

### Pre-testing

Xpert MTB/RIF pre-testing of five DTS from each stock was conducted to evaluate DTS accuracy and precision on a limited scale prior to preparing large numbers of PT samples. Dried tube specimens were prepared by diluting an aliquot of each stock suspension 1:10 with in-house prepared, sterile saline, adding food grade dye at a concentration of 0.1% (volume/volume) to each dilution, and transferring 100 µL of each stock dilution into five 4 mL cryovials. Cryovials were left uncapped in a Class II biosafety cabinet (BSC) until all liquid evaporated and aliquots were visibly dry. Dried tube specimen samples were then capped and stored in the dark at room temperature until tested.

**FIGURE 2:**
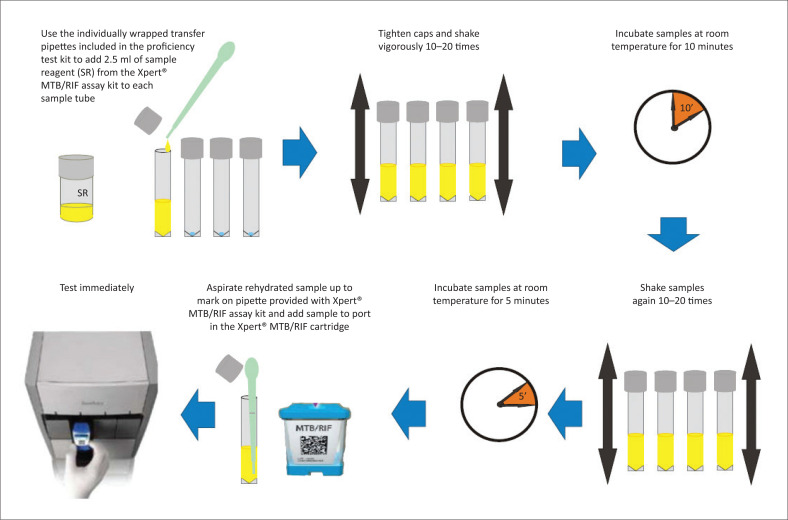
Testing dried tube specimens using Xpert MTB/RIF, 2013–2015, United States Centers for Disease Control and Prevention (Atlanta, Georgia, United States).

Dried tube specimens were rehydrated with 2.5 mL of SR, shaken vigorously 20 times and incubated at room temperature for 15 min with additional shaking repeated after 10 min (Figure 2). The Xpert MTB/RIF cartridges were inoculated with approximately 2.0 mL of rehydrated samples using the transfer pipette provided with the kit, and tested immediately on a GeneXpert IV or GeneXpert VIII using GeneXpert DX software version 4.0 (Cepheid, Sunnyvale, California, United States), according to manufacturer’s instructions.^17^ Dried tube specimens prepared in 2013 were tested with Xpert MTB/RIF Assay G4 Research Use Only cartridges. Dried tube specimens prepared in 2014 and 2015 were tested with Food and Drug Administration-cleared Xpert MTB/RIF US In Vitro Diagnostic (IVD) cartridges.

To ensure that DTS yielded accurate Xpert MTB/RIF results, *M. tuberculosis* detection and rifampicin resistance results from DTS pre-testing were verified against the expected Xpert MTB/RIF results for the parent strain (as determined by prior phenotypic and genotypic testing). Results were considered acceptable if the qualitative *M. tuberculosis* detection and rifampicin resistance results matched the expected results for the parent strain for all five pre-tested DTS from the stock. Precision was evaluated to verify uniform suspension of the organism in the stock using the cycle threshold (Ct) standard deviation (SD). In line with previous studies, Probe A was selected for most analysis as it was the first probe to reach the detection threshold.^18^ The SDs of probe A Ct values were calculated for all DTS and compared between different samples within each panel. Probe C Ct values were used when probe A did not bind. The SD for the cartridge internal specimen processing control (SPC) Ct was also calculated for all samples and panels to investigate whether inherent Xpert MTB/RIF cartridge variability contributed to variation in DTS Ct values. To ensure that adequate amounts of inactivated organism were present in the DTS and that the integrity of DNA was not compromised during inactivation, the average Xpert MTB/RIF semi-quantitative result (i.e. the category assigned to the amount of *M. tuberculosis* DNA based on the Ct of the first probe detected) was required to fall in the medium (Ct = 16–22) to low (Ct = 22–28) range.

### Stock selection and dried tube specimen panel validation

The five most precise and accurate stocks (i.e. the stocks with the lowest Ct SD and lowest mean Ct with DTS all yielding expected test results during pre-testing) were selected to aliquot DTS for use in the PT programme. Each Xpert MTB/RIF PT panel included one DTS per stock, for a total of five samples. Eight unique panels were prepared for use in the PT programme from 2013 (2013-A, 2013-B, 2013-C, and 2013-D) to 2015 (2014-A, 2015-A, 2015-B, and 2015-C) as described above. A strict BSC cleaning protocol (1% sodium hypochlorite followed by 70% ethanol rinse of all inner walls, sash, work surface and under work surface) was employed before aliquoting each panel. In most cases, only one sample was dried at a time. In situations where more than one sample was dried in the same BSC at the same time, all DTS had the same expected result. An aliquoting order was routinely followed: (1) ‘Tuberculosis Not Detected’ (non-tuberculous mycobacteria) samples were first aliquoted, then dried, capped, and removed; (2) ‘Tuberculosis Detected, Rifampicin Resistance Not Detected’ samples followed; and finally (3) ‘Tuberculosis Detected, Rifampicin Resistance Detected’ samples were aliquoted last. No culture manipulation was performed in the BSC while DTS samples were drying. Once prepared, panels were validated by confirming accurate and precise test results for 10% of the DTS produced using the same methodologies outlined above. Ten DTS from each stock underwent validation for panel 2013-A (50 total), 18 for panel 2013-B (90 total), 25 for panels 2013-C, 2013-D, and 2014-A (125 total per panel), 50 for panels 2015-A and 2015-B (250 total per panel), and 55 for panel 2015-C (275 total).

## Results

Across all eight panels, the mean Ct values for probe A (or probe C when probe A did not bind) ranged from 19.3 to 26.2, which were within the acceptable Xpert MTB/RIF semi-quantitative range of medium to low (data not shown). The SPC Ct ranged from 0.0 to 40.8 across all eight panels with a mean Ct range of 23.8–27.8. When panel validation results were compared with the expected Xpert MTB/RIF results for the parent strain, DTS tested from panels 2013-A through 2015-C ranged from 96.0% to 98.5% concordant (Table 1). Only three (0.2%) of the 1290 DTS tested were discordant, and of those, two were false negative and one was false positive. A total of eight (0.6%) DTS samples, including four from 2013-B, three from 2013-C, and one from 2013-D, yielded indeterminate rifampicin results. When examining Cts for all discordant and rifampicin-indeterminate results, we found that the Cts for the test cartridge SPC were consistently high (35.3–38.2) with the exception of one discordant result from 2015-B (25.7).

**TABLE 1:** Accuracy of dried tube specimen Xpert MTB/RIF test results collected during panel validation, 2013–2015, United States Centers for Disease Control and Prevention (Atlanta, Georgia, United States).

PT Panel	Concordant		Discordant		RIF Indeterminate		Error		Total	
	*n*	%	*n*	%	*n*	%	*n*	%	*n*	%
2013-A	49	98.0	0	0.0	0	0.0	1	2.0	50	100
2013-B	81	90.0	1†	1.1	4	4.4	4	4.4	90	100
2013-C	121	96.8	1	0.8	3	2.4	0	0.0	125	100
2013-D	122	97.6	0	0.0	1	0.8	2	1.6	125	100
2014-A	120	96.0	0	0.0	0	0.0	5	4.0	125	100
2015-A	241	96.4	0	0.0	0	0.0	9	3.6	250	100
2015-B	244	97.6	1	0.4	0	0.0	5	2.0	250	100
2015-C	271	98.5	0	0.0	0	0.0	4	1.5	275	100

**Total**	**1249**	**96.8**	**3†**	**0.2**	**8**	**0.6**	**30**	**2.3**	**1290**	**100**

PT, proficiency testing; RIF, rifampicin.

† , Exact proportions are provided; totals may not add up to 100% because of unrepresented fractions.

A total of 30 errors were encountered while testing the 1290 DTS, which resulted in an overall error rate of 2.3% (Table 2). This rate ranged from 0.0% to 4.4% across panels (Table 1). Errors encompassed six different error codes (Table 2). No ‘Invalid’ or ‘No Result’ results were observed.

**TABLE 2:** Errors received during validation of dried tube specimens for 2013–2015 proficiency testing panels, 2013–2015, United States Centers for Disease Control and Prevention (Atlanta, Georgia, United States).

Error code	Error description†	Number detected	
		*n*	%
2005	Motion of the syringe drive was not detected.	3	10
2014	The digital temperature reading of n for Thermistor A/Thermistor B/Ambient Thermistor/Optic Thermistor was not within the acceptable range of m1 to m2.	1	3.3
2037	Cartridge integrity test failed at valve position n.	1	3.3
5006/5007	X probe check failed.	24	80
5011	Signal loss detected in the amplification curve for analyte [x].	1‡	3.3

**Total errors**		**30**	**-**

† , Error descriptions are from GeneXpert Dx 4.0 Operator Manual; 300-7607. Rev2010.

‡ , Exact proportions are provided; totals may not add up to 100% because of unrepresented fractions.

**FIGURE 3:**
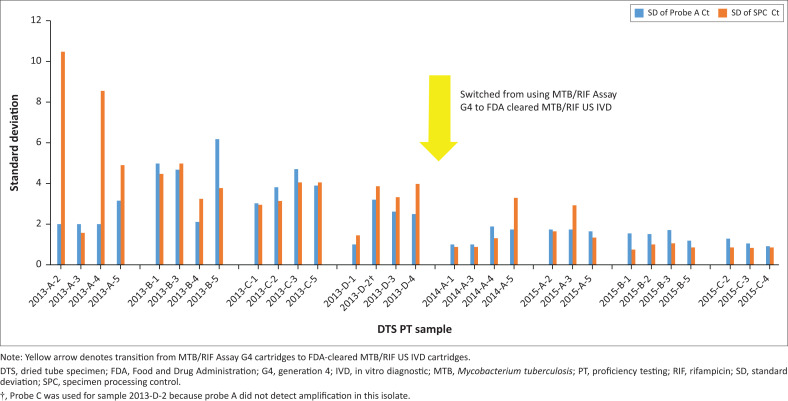
Standard deviations of Probe A and specimen processing control cycle threshold values for dried tube specimens containing *Mycobacterium tuberculosis* complex tested during panel validation, 2013–2015, United States Centers for Disease Control and Prevention (Atlanta, Georgia, United States).

While DTS samples were found to be accurate (96.8% concordance overall), sample precision varied, with Ct SD values ranging from 0.9 to 6.2 (Figure 3). The SD of the cartridge SPC also varied among DTS PT samples, ranging from 0.7 to 10.5. We observed higher variability in Probe A Ct (SD: 1.0–6.2) and SPC Ct values (SD: 1.4–10.5) for panels produced in 2013 and tested with Xpert MTB/RIF Assay G4 cartridges than for those produced in 2014 (1.0–1.9; 0.9–3.3) and 2015 (0.9–1.7; 0.7–2.9), which were tested with Food and Drug Administration-cleared Xpert MTB/RIF US IVD cartridges.

## Discussion

The DTS method was originally developed for HIV rapid test PT and subsequently used to produce PT panels for other assays such as syphilis, HIV viral load and malaria rapid test, with good results.^15,19,20,21^ This study demonstrates that an accurate and precise *M. tuberculosis* PT panel for the Xpert MTB/RIF assay can also be produced using the DTS technique. The panel validation process verifies the accuracy and precision of each DTS panel produced and adheres to Clinical and Laboratory Standards Institute recommendations for characterising molecular PT materials.^12^ The DTS panels produced according to this methodology were accurate, achieving 96.8% test result concordance with parental stocks for the detection of *M. tuberculosis* and rifampicin resistance. Interestingly, the discordant and indeterminate samples, with the exception of the 2015-B false negative discordant sample, had SPC Ct values above 35. A previous quantitative assessment by Blakemore et al. found that Xpert MTB/RIF assay results with SPC Ct values above 34 could be quantified inaccurately.^22^ Thus, it is possible that the majority (2 of 3) of the discordant results and all 8 of the RIF indeterminate results in the DTS panel evaluations could be due to factors inherent to the Xpert MTB/RIF assay cartridges. The overall error rate was relatively low (2.3%), and no trend in error rates was observed over the study period. Eighty percent of the errors observed were due to error codes 5006 and 5007 (probe check failure). These errors often are associated with the incorrect sample volume added to the cartridge, incorrect filling or bubbles in the cartridge reaction tube, or probe integrity issues.^10^ The remaining errors (codes 2005, 2014, 2037 and 5011) are associated with instrument or cartridge performance.

Additionally, the DTS method delivers a consistent amount of *M. tuberculosis* to each sample, such that testing of DTS prepared from the same stock yielded Probe A Ct values with an SD similar to the SD of the SPC internal control Ct values in most cases. There is no current standard for molecular PT variability. However, the standard deviations observed for DTS produced in 2014 and 2015 and tested with Food and Drug Administration-cleared Xpert MTB/RIF US IVD cartridges are in line with those seen by Scott et al. using a similar inactivation technique.^18^

Xpert MTB/RIF SR has been shown to effectively inactivate mycobacteria while leaving the cells intact.^18^ Although this evaluation did not assess the cell structure post-inactivation, other investigators confirmed the presence of whole bacilli following SR-mediated inactivation of *M. tuberculosis* using flow cytometry.^18^ Inoculation of DTS stock suspensions into MGIT culture provides confirmation of inactivation prior to distribution of DTS. Furthermore, the incubation of MGIT culture for 4–6 days post-positivity did not adversely affect inactivation with SR and was found to consistently yield DTS samples with the desired *M. tuberculosis* DNA concentration for DTS preparation and testing.

While the accuracy and precision of DTS are similar to those reported for other PT panels, the simplicity of matrix preparation and feasibility for transfer to low-resource settings set the DTS Xpert MTB/RIF PT panel apart.^13^ Additionally, since the procedure is similar to specimen processing for TB culture, much of the laboratory expertise, technical skill, equipment (e.g., Class II BSC, safety centrifuge, BACTEC MGIT 960 or 320 instrument, vortex, and an auxiliary incubator), and consumables necessary for DTS preparation already exist in tuberculosis culture laboratories in resource-limited countries. Many national tuberculosis reference laboratories in these settings have implemented BACTEC MGIT 960 culture and drug susceptibility testing in recent years, making MGIT 7 mL tube media the most common liquid media for *M. tuberculosis* culture and detection.^23^ Lastly, since DTS are prepared within the tubes utilised for sample rehydration, testing of DTS does not require additional laboratory supplies.

Not only are expertise, equipment, and supplies readily available, but DTS are also estimated to be low cost. Regarding transport, Parekh et al. reported that the use of DTS for HIV PT eliminates the need for cold chain and results in a significant reduction in shipping costs.^15^ Reagent costs are also low, as approximately 500 DTS can be made from a single inactivated MGIT culture. It is therefore possible for one culture laboratory, such as a national tuberculosis reference laboratory, to produce and validate enough DTS to provide PT material for all testing sites in a country. The DTS method can thus provide resource-limited countries with a suitable PT material to create and manage their own sustainable Xpert MTB/RIF PT programme. External quality assessment using DTS can serve as an important component of a laboratory QA programme, which is necessary to ensure that patients receive accurate and reliable diagnostic testing services.^11^

### Challenges and limitations

The primary challenge associated with producing DTS was variability between Ct values from separate aliquots of the same dilution. However, the inherent cartridge-to-cartridge variability in Ct values that was observed when comparing the SD of probe A to the SD of the SPC likely plays a role in the variability observed in DTS Ct values. After the Xpert MTB/RIF assay gained Food and Drug Administration clearance in 2013, we transitioned from using Xpert MTB/RIF Assay G4 Research Use Only cartridges in 2013 to Xpert MTB/RIF US IVD cartridges in 2014 and 2015. The range of SDs of probe A Ct values decreased from 1.0–6.2 in 2013 to 0.9–1.9 in 2014–2015. This decrease further suggests that the Ct value variability and high SD values observed were likely due to inconsistencies between cartridges. However, the variability of DTS could also be due to mycobacteria (particularly *M. tuberculosis*) clumping in MGIT cultures, leading to an uneven DTS stock suspension and difficulty in achieving equal numbers of bacilli in each aliquot. Additional evaluations are underway to improve both the accuracy and precision of DTS.

Another limitation of using the DTS technique for PT is that the DTS sample type (inactivated, dried mycobacteria) does not resemble the most commonly tested clinical sample type, sputum. While the use of DTS for PT does not allow for the complete evaluation of all the procedural steps or potential pitfalls involved in Xpert MTB/RIF testing of sputum, it could be an adequate substitute. For example, creating lyophilised, *M. tuberculosis*-spiked sputum samples would involve the purchase and maintenance of expensive equipment, such as lyophilisers, and increase biosafety risk for laboratorians. Alternately, the use of liquid samples increases biohazard risk to both shipping and laboratory personnel and increases transportation costs. Therefore, the safety and cost-saving benefits of DTS may outweigh potential disadvantages associated with assessing proficiency of Xpert MTB/RIF testing using dried, inactivated *M. tuberculosis* in resource-limited settings.

### Recommendations and next steps

We are continuing to study and refine the technique for producing DTS, including the investigation of heat inactivation of *M. tuberculosis* for improved biosafety, stability and sample accuracy and precision. Since development of this novel PT technology, a voluntary Xpert MTB/RIF DTS-based PT programme has been rolled out to 26 countries and United States-affiliated territories, as described in ‘A Global Proficiency Testing Program for Xpert MTB/RIF using Dried Tube Specimens’ by Klein et al.^24^ In addition, in 2016 we began the transfer of DTS technology to countries as part of a PT package that includes feedback and corrective actions to sustainably improve the quality of international diagnostic testing services. In 2019, the scope of the PT programme was increased to include the Xpert MRB/RIF Ultra assay (Cepheid, Sunnyvale, California, United States) where the same panel prepared for Xpert MTB/RIF PT was also successfully validated for use as PT for the Xpert MTB/RIF Ultra assay.

### Conclusion

Proficiency testing for the Xpert MTB/RIF assay, as part of a comprehensive QA programme, should be a priority for every national tuberculosis programme. The DTS technique creates accurate, precise, and safe PT panels. Designing this preparation procedure around the availability of equipment and reagents in national tuberculosis reference laboratories in resource-limited countries promotes the transfer of DTS technology to both national and regional programmes and assists countries in implementing consistent, sustainable EQA programmes.
